# Strategies to Implement Knee Osteoarthritis Guidelines in Switzerland

**DOI:** 10.3389/phrs.2026.1609071

**Published:** 2026-03-12

**Authors:** Céline Moetteli-Graf, Karin Niedermann

**Affiliations:** 1 School of Health Sciences, Zurich Universiy of Applied Sciences, Winterthur, Switzerland; 2 Faculty of Medicine, University of Zurich, Zurich, Switzerland; 3 Swiss School of Public Health, Zurich, Switzerland

**Keywords:** chronic care, guideline adherence, knee osteoarthritis, model of care, patient care management

## Abstract

**Background:**

Knee osteoarthritis (KOA) is a common chronic disease in Switzerland, associated with high socioeconomic costs and increasing prevalence due to aging and other risk factors. International guidelines recommend a stepped approach focusing on exercise, education, and weight management; however, implementation remains inconsistent. Switzerland has one of the highest knee replacement rates among OECD countries, suggesting potential overuse and indicating an evidence–performance gap in KOA management.

**Analysis:**

Current efforts promote coordinated, patient-centered care. A repeated stakeholder dialogue in 2024 with representatives of medical and physiotherapy associations, patient organizations, health insurers, and researchers identified challenges: variation in patient pathways across providers; non-evidence-based treatment sequencing with premature escalation to specialist care; and misalignment of expectations between patients and providers.

**Policy Options:**

Proposed strategies include: (1) establishing a national Model of Care based on a consensus treatment framework; (2) strengthening patient health literacy through targeted education; and (3) facilitating patient navigation through effective communication and shared decision-making.

**Conclusion:**

Closing the evidence–performance gap requires collaboration among healthcare providers to improve outcomes, reduce inappropriate care, and support coordinated patient-centered KOA management in Switzerland.

## Background

Osteoarthritis is the most common chronic joint disease, characterized by pain and loss of function [1]. Knee joints are particularly affected, with a prevalence of 12.4% in Switzerland that increases with age, impacting nearly one-third of individuals over the age of 75 [2]. This imposes a significant individual and socioeconomic burden. In 2017, the direct medical costs of osteoarthritis in Switzerland amounted to 1.5 billion CHF, representing 1.8% of total annual healthcare expenditures [3]. With an ageing population and rising obesity, alongside other risk factors such as previous joint injury or genetic predisposition, the burden of osteoarthritis is becoming a growing problem [4, 5]. According to Global Burden of Disease projections, the number of knee osteoarthritis (KOA) cases is expected to increase by around 75% by 2050 in high-income countries, mainly driven by population ageing [6]. Consistent with this trend, European projections estimate an increase of approximately 50% in total knee arthroplasty (TKA) volumes over the coming decades, posing a major challenge for future healthcare systems [7–9].

International clinical guidelines consistently recommend a patient-centered, stepped approach to managing KOA [10–12]. The core elements focus on conservative, primarily non-pharmacological treatments, including structured exercise, education, and, if needed, weight reduction, supported by basic pain medication, primarily topical or oral nonsteroidal anti-inflammatory drugs (NSAIDs). If options are exhausted, surgical interventions may be considered. Despite strong and consistent scientific evidence, the guideline recommendations are internationally not systematically implemented in practice and there appears to exist a so-called “evidence-performance gap”.

In an international review of osteoarthritis care, Basedow et al. [13] examined quality indicators across diagnosis, non-drug and drug treatments, and surgery. They found overall low quality of care, with the greatest gaps in the systematic assessment of pain and function as well as in the consistent use of non-drug treatments such as exercise and education. A recent survey among general practitioners, rheumatologists, and orthopedic surgeons in Switzerland revealed that the doctors estimated to refer around 54% of their patients with KOA to a specific exercise program. Barriers to prescribing exercise included patients’ expectations and lack of interest, as well as clinicians’ own clinical experiences [14]. In Switzerland, where physiotherapy requires a medical prescription, physicians act as gatekeepers to exercise therapy, making referral rates a key indicator of access to non-pharmacological care. For patients, limited access to recommended conservative care can translate into avoidable symptom progression and earlier loss of mobility.

While non-pharmacological management of KOA appears to be underused, there are indications of an overuse of surgical interventions. Switzerland ranks highest among OECD countries in knee replacement surgeries per 100,000 inhabitants [15]. Although the number of TKA alone does not reveal whether these surgeries are appropriate or overused, research highlights the significant influence of economic factors on surgery rates. Higher rates of TKA have been associated with countries that have greater economic resources, including a higher gross domestic product (GDP) and higher public healthcare expenditure. In contrast, a stronger gatekeeping role by general practitioners is associated with lower surgery rates [16–18]. This suggests that the number of surgeries performed may not solely reflect the healthcare need of the population, but is also influenced by healthcare financing and available resources. High and potentially inappropriate surgery rates not only expose patients to avoidable risks but may also increase the number of individuals who suffer from postoperative complications.

In Switzerland, surgery rates show modest regional variability, indicating a consistently low threshold for performing TKAs across regions. However, one-third of this variation remains unexplained and, according to the study authors, may partly reflect differences in physicians’ beliefs and attitudes toward joint arthroplasty [19]. The Swiss Healthcare Atlas confirms the moderate regional variation in initial knee replacements. A concordance coefficient of 0.73 indicates that these regional patterns have remained relatively stable over time [20]. This suggests that the differences in surgery rates are systematic rather than random, pointing to structural factors such as regional differences in healthcare supply, referral pathways, and access to orthopedic services as contributors to the observed regional variability.

Overall, these findings indicate a substantial mismatch between evidence-based recommendations, clinical practice, and health system incentives in KOA care, underscoring the need for more coordinated and structured approaches to management.

## Analysis

To understand the policy context of KOA care, we reviewed Swiss strategies on chronic disease management, which increasingly emphasize coordinated, evidence-based care. In the Health2030 Strategy, the Federal Council highlighted overuse, underuse, and misuse of services as risks to both quality and sustainability of care, calling for stronger coordination and for citizens to be empowered to make informed choices [21]. The subsequent Health2040 Action Plan translates this call into a patient-centered health network that emphasizes collaboration across sectors, better navigation of patients across services, and equitable access. These issues are directly relevant to KOA, where care remains fragmented and navigation inconsistent [22]. More recently, the “Agenda Grundversorgung” has placed continuous and coordinated management of chronically ill patients at the center of primary care reform, reinforcing the relevance of KOA as a long-term condition requiring structured pathways [23]. Together, these policy approaches signal an increasing recognition of the need for more structured and coordinated care for chronic diseases, including KOA. However, they have so far not resulted in a nationally standardized approach to KOA management. In fact, the GLA:D program represents a national initiative to implement the guideline recommendations of exercise and education as first-line treatment; however, it is not yet embedded in a consistent treatment pathway.

To address these challenges, a repeated stakeholder dialogue was conducted in 2024. Stakeholders were selected to represent key perspectives on KOA management in Switzerland from medical societies, the national physiotherapy association, two organizations of Swiss health insurers, research institutions with expertise in health economics, physiotherapy, and implementation science, as well as the Swiss League Against Rheumatism as a patient organization. Each institution was invited to mandate an official representative. Further details on the dialogue design, participating stakeholder groups, and thematic focus are documented in a publicly available summary report of the stakeholder dialogue (available in German) [24].

The process began with a structured dialogue to identify consensus and disagreement on KOA management, followed by four thematic meetings between May and December 2024. While the initial dialogue followed a formal structure, the follow-up sessions encouraged open exchange, collaborative problem-solving, and the development of actionable strategies.

The stakeholder dialogue collectively highlighted three core challenges in KOA management in Switzerland. 1) There is no standardized approach for navigating patients through the healthcare system. Despite the availability of international clinical guidelines, patient pathways remain inconsistent, often shaped by the individual preferences of patients and healthcare professionals. This lack of clarity on decision criteria leads to fragmented and uncoordinated care. 2) The timing and sequencing of interventions often do not follow a stepwise, evidence-based escalation. Conservative treatments are delayed or omitted, while escalation to specialist care or surgery occurs too early or without sufficient use of first-line options. 3) Healthcare providers face difficulties in managing expectations and motivating patients to engage in conservative care. Patient acceptance of nonsurgical treatments appeared low. A Swiss survey among GPs, rheumatologists, and orthopedic surgeons confirmed these challenges, identifying patients’ disinterest and demand-driven referrals as major barriers to recommending exercise programs [14].

## Policy Options

**FIGURE 1:**
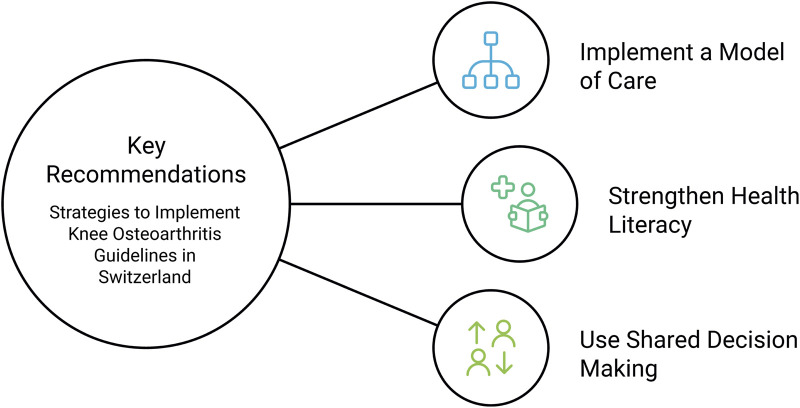
Key recommendations for implementing knee osteoarthritis guidelines in Switzerland (policy framework, Switzerland, 2026).

This Policy Brief builds on the results of this repeated dialogue, complemented by evidence from the scientific literature, to formulate three recommendations as seen in Figure 1.

### Implement a Model of Care Based on Consensus-Based Treatment Framework Grounded in International Guidelines to Promote Multidisciplinary and Evidence-Based Care

Implementing a Model of Care (MoC) offers a structured solution to the challenges identified in the stakeholder dialogues. A MoC can be defined as an “evidence-informed strategy, framework or pathway that outlines the optimal manner in which condition specific care should be delivered to consumers within a local health system” [25]. Its aim is to explicitly translate evidence-based guidelines into practice and facilitating their implementation. It provides guidance on delivering “the right care, at the right time, by the right team, in the right place, using the right resources” [25] - an approach increasingly adopted internationally for osteoarthritis management. A scoping review identified 37 international examples of MoCs for KOA across 13 mostly high-income countries [26]. While the structure of these models varies, they all incorporate at least one of the core guideline recommendations: self-management, education, exercise, and/or dietary weight management. Examples include the AktivA program in Norway, which emphasizes physiotherapy-led education and exercise with demonstrated long-term benefits; the BART program in the Netherlands, which uses a stepped-care approach to ensure conservative treatments are prioritized before specialist referral; and the Victorian Model of Care in Australia, which highlights structured care pathways, multidisciplinary collaboration, and patient education. Despite differences in design, common elements across these models include GP-led care, structured referral pathways, and multidisciplinary teamwork. Evidence from stepped-care models suggests that structured pathways improve the uptake of conservative treatments and are associated with a more appropriate use of secondary care services [27]. Data from the Norwegian AktivA implementation model further demonstrate patient-level benefits of such conservative care, including a clinically relevant reduction in pain (NRS 5.2 to 4.1), sustained improvements in disease-specific quality of life (KOOS/HOOS +8–9 points), and a reduction in physical inactivity from 43% to 22%, with effects maintained over 2 years [28].

For Switzerland, a MoC for KOA could build around a consensus-based treatment framework. Such a framework would translate international recommendations into a practical structure that supports decision-making within a stepped-care model. A visual decision tree could support providers in guiding patients, while an adapted lay version would serve as an educational tool to strengthen patient understanding. Embedding the framework into digital platforms or electronic health records would ensure accessibility and promote consistent communication across providers. The choice and design of the implementation strategies will be guided by a context analysis to identify barriers and facilitators. GLA:D could serve as the standardized program for exercise therapy and patient education within a Swiss MoC with a broader scope, coordinating the full patient pathway from early conservative management to orthopedic evaluation and surgical care.

Experience from the implementation of a national diabetes MoC shows that barriers occur across system levels, including unclear governance and lack of sustainable financing for coordinated care, limited integration into organizational care pathways, and constrained capacity of individual providers to adopt new roles within routine practice [29]. Addressing these barriers requires coordinated responsibilities across system levels: the federal level can provide funding frameworks and supportive regulatory conditions, professional associations can lead consensus-building and the development of the treatment framework, insurers can align reimbursement and financial incentives, and cantons play a key role in integrating the MoC into regional care structures and service planning. Implementation requires targeted resources but may improve efficiency. A national MoC can serve as the foundation, supported by health literacy and SDM strategies.

Reflecting this need, the final stakeholder meeting led to a joint decision to prepare a project proposal aimed at implementing KOA guidelines in Switzerland. This initiative underlines the strong commitment of all parties to improve the quality of care.

### Strengthen the Health Literacy of Individuals With KOA Through Targeted Education Initiatives

An Australian study on patient beliefs about knee osteoarthritis identified widespread misconceptions, with many patients viewing the condition as “wear and tear” or “bone-on-bone” degeneration. These beliefs were associated with fear of movement, reluctance to engage in physiotherapy or exercise, and a perception of surgery as the only viable solution [30]. Negative beliefs including movement-related fears in musculoskeletal disorders are associated with lower health literacy [31]. Low health literacy negatively affects health behaviors, health outcomes, and healthcare utilization. Health literacy is the ability to access, understand, appraise, remember and apply health information to make informed decisions. It encompasses multiple dimensions, including feeling understood and supported by healthcare providers, the ability to actively engage in care, and to navigate the healthcare system [32]. In Switzerland, 49% of the population reports frequent difficulties with health information. Those most affected include individuals with financial hardships, language barriers, low social support, chronic diseases, older age, or residence in rural areas [33]. To ensure equitable access to evidence-based KOA management, targeted health literacy initiatives are needed.

One promising approach is the Ophelia (Optimizing Health Literacy and Access) project, which has been applied internationally to improve health outcomes and equity by addressing health literacy barriers. The framework combines assessment of local needs, co-design of tailored interventions, and systematic evaluation. For example, in a Danish cardiac rehabilitation program, Ophelia identified patient difficulties in understanding rehabilitation guidelines. In response, simplified materials and personalized counseling were developed, leading to greater engagement and adherence [34]. Applied to KOA management in Switzerland, this structured process offers a way to systematically identify barriers related to understanding, engagement, and system navigation, and to translate them into locally adapted solutions that support shared decision-making and patient navigation across the care pathway. Many patients lack awareness of conservative treatments and hold misconceptions about disease progression, leading to unnecessary specialist visits and underuse of nonsurgical interventions. Adapting the Ophelia model would involve assessing specific patient needs, developing tailored tools and educational materials, and integrating these interventions into primary care and physiotherapy settings. This could empower patients to actively participate in their treatment, reduce the burden on specialists, and enhance equity in care. Strengthening health literacy in this way would contribute to more efficient and evidence-based KOA management within the Swiss healthcare system.

Effective implementation will require interventions that are co-designed with patients and adapted to the needs of diverse population groups. Information should be made available in simple and visual formats, in multiple languages, and embedded in routine encounters in general practice and physiotherapy. Patient organizations can support co-design, outreach, and dissemination to ensure materials are relevant and accessible. Clear governance and prioritization are also needed: federal authorities can anchor health literacy in national NCD strategies, cantons can integrate initiatives into existing prevention structures, insurers can reimburse counselling and education, and professional associations can promote health-literate communication in routine care. Prioritizing co-designed interventions may achieve the greatest reach and equity.

### Facilitate Patient Navigation Through the Treatment Framework Using Effective Communication and Shared Decision-Making Practices

While strengthening health literacy can help align patient expectations with evidence-based care, effective communication by healthcare professionals is equally crucial. Clear guidance, shared decision-making (SDM), and transparent discussions about treatment options can improve patient understanding and acceptance of non-surgical interventions. SDM specifically addresses belief and knowledge imbalances that often drive inappropriate healthcare demand. It involves healthcare professionals and patients collaborating on healthcare decisions, considering the best available evidence and the patients’ values and preferences. By supporting patients to better understand and reflect on available options, SDM can also reinforce key dimensions of health literacy and complement structured approaches such as the mentioned Ophelia process. Informed, patient-centered decisions are associated with better health outcomes and higher satisfaction for both KOA patients and healthcare professionals [35, 36].

In southwest England, an SDM tool to support patients with KOA in making informed treatment choices was implemented in 2022. The tool is a locally adapted shared decision-making (SDM) tool for KOA, as described by Turnbull et al. [37]. It provides evidence-based information on available treatment options across all disease stages, guiding discussions between patients and clinicians. Available in both digital and paper formats, it aims to enhance patient engagement and encourage conservative management before considering surgery. The tool was particularly valued for improving patient understanding of KOA management, aligning expectations with value-based care, and promoting non-surgical options. Implementing SDM in a similarly systematic manner in Switzerland could improve patient engagement. A structured SDM approach could improve patient satisfaction by enhancing communication and empowering individuals to take an active role in their care. When patients are well-informed and engaged, they are more likely to adhere to treatment plans, leading to better health outcomes. Additionally, SDM promotes greater equity in healthcare by ensuring that all patients, regardless of health literacy, can navigate the treatment framework, fostering more consistent and inclusive KOA management. However, challenges must be addressed. Healthcare providers may require additional training in SDM and effective communication. Furthermore, longer consultations could create time constraints and increase the workload of healthcare professionals, necessitating strategies to integrate SDM efficiently into clinical practice. Training is needed to prepare GPs, orthopedic surgeons, rheumatologists, and physiotherapists to apply SDM in daily care. Federal and cantonal authorities can support SDM competencies in education frameworks, universities can integrate SDM into curricula, and professional associations can promote SDM standards and training. Embedding SDM tools into electronic records can support uptake, while insurer incentives may help compensate for longer consultations.

## Conclusion

KOA poses a growing challenge for the Swiss healthcare system. Evidence-based conservative treatments remain underused, while surgical care seems to be overused, resulting in an evidence–performance gap. The repeated stakeholder highlighted three core challenges: inconsistent patient navigation, non-evidence based timing and sequencing of interventions with premature escalation of care and difficulties in aligning expectations between patients and providers. Closing these gaps requires a joint effort and collaboration of healthcare providers across disciplines. A national MoC, built on a consensus-based treatment framework, could establish structured pathways and reduce fragmentation. Strengthening patient health literacy through targeted, co-designed education would empower individuals to engage in conservative management. At the same time, systematically embedding shared decision-making into consultations and treatment discussions would align care with both the best available evidence and patient values. Together, these measures could improve outcomes, reduce inappropriate care, and support coordinated, patient-centered KOA management in Switzerland. As an immediate next step, a national multi-stakeholder initiative should advance a Swiss KOA MoC to strengthen interprofessional coordination and communication and to reinforce the paradigm of “conservative care before surgery”. This process should be led in collaboration with relevant medical societies and professional associations, alongside early engagement of policymakers to foster long-term system integration and sustainable financing.
